# Electrons and X-rays for diffraction and imaging

**DOI:** 10.1107/S2059798326002056

**Published:** 2026-04-07

**Authors:** Colin Nave, Pedro Nunes, Alistair Siebert

**Affiliations:** a Diamond Light Source Didcot United Kingdom; University of Oxford, United Kingdom

**Keywords:** X-ray crystallography, electron crystallography, electron imaging, X-ray imaging, biology

## Abstract

A comparison is provided between the use of electrons and X-rays for collecting diffraction data from small protein crystals and imaging data from cells and tissues. The paper contains a review element written to enable an understanding of the relevant properties of electrons by those (including one of the authors) more used to X-ray imaging and diffraction. Radiation-damage mechanisms, sample thickness-dependent dose efficiency and energy-dependent scattering cross sections are discussed, together with contrast mechanisms in electron and X-ray imaging. Crossover points are calculated for diffraction from crystals where electrons and X-rays could yield equivalent data quality in the presence of radiation damage. Increasing the electron energy from 300 to 1000 keV results in a ∼43% rise in the electron/X-ray crossover point. However, the maximum information coefficient (useful signal/absorbed dose) for electrons alone occurs for a 250 nm crystal examined at around 800 keV. The impact of inelastic scattering on electron imaging and diffraction is examined, including its role in coherence loss, Bragg spot broadening and background elevation. The possibilities are investigated for locating regions of interest using X-raysfor subsequent higher resolution imaging using electrons. For locating a 30 nm diameter protein or virus, the required X-ray dose would be much less than the tolerable dose for electron imaging at 0.5 or 0.25 nm. Overall, these findings are relevant for imaging at different length scales while minimizing dose-induced structural damage.

## Abstract 2

The use of X-rays and electrons for diffraction and imaging of soft biological samples is compared.

## 1. Introduction

The improvements in both electron and X-ray imaging have meant that a combination of the two methods is becoming increasingly attractive for the examination of biological materials, such as proteins, cells and tissues, across different length scales. X-rays are also used for diffraction from ever-smaller crystals using brighter X-ray sources, serial synchrotron crystallography (SSX) and serial femtosecond crystallo­graphy (SFX) on free-electron lasers. Simultaneously, electron diffraction is being developed to examine submicrometre protein crystals. Although the term diffraction is normally applied to crystals, diffraction-based imaging techniques such as coherent diffraction imaging (CDI) and ptychography are increasingly being applied to noncrystalline biological samples using electrons (Zhou *et al.*, 2020 ▸) and X-rays (Bosch *et al.*, 2025 ▸). Fundamental properties such as cross sections between the incident radiation and the sample are similar for diffraction from crystals and various types of imaging, so there is significant commonality between them, with the most significant difference being the amplification power given by the Bragg spots in the diffraction pattern of a crystal. The choice of whether to use electrons or X-rays, together with the optimum energy to use, is becoming an increasingly important issue. An important goal is to maximize the signal and minimize the dose. An understanding is required of the various mechanisms by which dose is absorbed in the sample, the effects of such a dose and the most efficient and noise-free way of collecting the data.

The paper is structured to cover these aspects for electron diffraction and imaging, and then for X-ray diffraction and imaging. A comparison of the two is then made to give guidance on the most appropriate method to use. This includes calculating the crossover points for specimen thickness where electrons and X-rays give a similar dose-to-signal ratio. The advantage of X-rays for thicker specimens means that it is possible to identify regions of interest in thick biological tissue for subsequent higher resolution examination by electron imaging. The X-ray dose required to identify such regions could damage the higher resolution details, so this aspect is discussed.

There are various models for radiation damage dependent on the material being studied, the resolution of interest and the type of damage of concern. The types of damage include the following.

(i) Small chemical changes which produce small atomic movements and do not necessarily lead to an observable increase in *B* factors in crystallography.
(ii) Significant random atomic displacements leading to an increase in *B* factors in crystallography. This can be reduced with the use of cryogenic temperatures. This is often referred to as global damage.
(iii) Specific atomic displacements producing observable structural changes, for example, breakage of disulfide bridges. These might not be observed at room temperature due to the increase in *B* factors. This is often termed specific damage.
(iv) Swelling of the sample, sometimes anisotropically. This can lead to changes in unit-cell dimensions at cryogenic and room temperatures.
(v) Similar macroscopic swelling of amorphous samples. In some cases, the local interactions (for example around 20 nm spacings between synapses in neurons) are still preserved.
(vi) Mass loss. Suppressed at cryotemperatures for frozen-hydrated specimens but eventually occurs.

Models for the loss of resolution as a function of dose have been developed for X-ray crystallography of proteins (see, for example, Howells *et al.*, 2009 ▸; Atakisi *et al.*, 2019 ▸) and the present paper is mainly based on these models.

## 2. Resolution and the Rose criterion

The contrast of a feature of interest in both electron and X-ray imaging is given by the difference in intensity between the feature and the surrounding background at a relevant spatial frequency. The Rose criterion (Rose, 1948 ▸) states that, for reliable detection, the contrast should be five times the standard deviation of the noise. However, a factor of three is often used, as in Egerton & Nave (2026 ▸). The Rose criterion is applicable to features with a well defined edge imaged against a uniform background. An example of such a feature in cell biology could be a virus particle imaged against the surrounding cytosol. In this case, the different parts of the feature scatter in phase at the resolution required to identify its presence. This is therefore within the coherent regime where the required fluence and dose varies with the inverse fourth power of the resolution as derived in Howells *et al.* (2009 ▸) and, using a different approach, in Nave (2018 ▸).

A Rose criterion of five times the standard deviation of the noise, when applied to the intensities, implies that to detect a feature above a background the difference electron density or Coulombic potential (between the feature and the surroundings) would have to scatter at least 25 photons or electrons. Starodub *et al.* (2008 ▸) suggested that only 6.25 photons would be needed to give a signal-to-noise ratio of 5 for the amplitudes, from which the electron densities are derived. This follows error propagation as the signal-to-noise ratio for the amplitude is twice the signal-to-noise ratio for the intensities, giving the required number of photons as 2.5^2^ assuming Poisson statistics. However, this applies to the required statistics for intensity measurements to obtain a defined signal-to-noise ratio in an electron-density (or Coulombic potential) map. It is a separate criterion from the application of the Rose criterion to calculate the signal-to-noise ratio of a feature observed being detected above the surroundings, as discussed in Nave (2023 ▸). The Rose criterion is not used for high-resolution protein structure determination as the features are imaged at spatial frequencies corresponding to van der Waals or covalent-bond distances and are not observed against a background. In these cases, Wilson statistics apply and the required fluence and dose varies with the inverse third power of the resolution (Shen *et al.*, 2004 ▸). The difference between the coherent and incoherent regimes was pointed out by Howells *et al.* (2009 ▸).

In many scientific disciplines, the word resolution applies to the instrument rather than a particular image obtained with it. The contrast-to-noise ratio for a feature of interest at the relevant spatial frequency is a more relevant metric (Nave, 2023 ▸). This applies, for example, to imaging cellular organelles, which might be planar, linear or globular and have different contrasts. However, for protein structure determination, the distribution of electron densities or Coulombic potential at the relevant spatial frequencies is similar for all proteins and the concept of resolution is useful for comparing different structures. A standard way of doing this is to use correlation coefficients between half data sets, and this approach is routine in the processing of diffraction data obtained from both electrons and X-rays.

## 3. Electron imaging and diffraction

In a key paper, Henderson (1995 ▸) compared electrons and X-rays (and neutrons) for single-particle structure determination of proteins, estimating that electrons give 500–1000 times more scattering than X-rays for the same dose (the exact number depends on the exact electron and X-ray energies and hence the cross sections used for the calculations; see Section 3.1). Since then, single-particle cryo-electron microscopy has become the method of choice for the structural determination of many proteins, particularly large macromolecular complexes, for which crystallization is often highly challenging.

The analysis in the present paper assumes that chromatic correction of inelastically scattered electrons (*i.e.* post-specimen chromatic correction) is not implemented as this is not at present routine, although progress has recently been made (Wu *et al.*, 2025 ▸). The role of inelastically scattered electrons combined with chromatic correction for *in situ* structure determination of proteins in thick specimens (for example biological cells) is also discussed in Dickerson *et al.* (2022 ▸). They estimate that the signal from thick specimens (in the 100–500 nm range) could be enhanced for samples imaged close to focus with an achromatic lens.

Our analysis of the crossover point for specimen thickness, where the signal from X-ray and electron crystallography is the same, assumes that energy filtering of inelastically scattered electrons can be applied. Explanatory diagrams of electron optics for energy filtering in electron diffraction can be found in Yang *et al.* (2022 ▸). The benefits of electron energy filtering in protein crystallography are described in Yonekura *et al.* (2019 ▸) and a review of electron crystallography of proteins is given in Clabbers *et al.* (2022 ▸).

The optimum electron energy for single-particle TEM has been discussed by Peet *et al.* (2019 ▸), including the derivation of an information coefficient *z,*
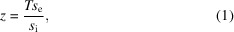
where *s*_e_ and *s*_i_ are the cross sections for elastic and inelastic scattering and *T* is the transmission. The transmission is given approximately by exp(−*t*/λ), where *t* is the specimen thickness and λ is the total mean free path (MFP) for electrons, estimated, for ice, as 314 nm at 300 keV. The information coefficient is maximized for high values of elastic scattering cross sections which contribute to the signal and for low values of inelastic scattering which deposits energy in the sample. The transmission loss accounts for both elastic and inelastic scattering, which weakens the direct beam, especially for thick samples. This construct assumes that inelastically scattered electrons are no longer focused at the image plane and do not contribute to image formation. As mentioned previously, recent work by Dickerson *et al.* (2022 ▸) and Wu *et al.* (2025 ▸) discusses and demonstrates the coherence loss by inelastic scattering of electrons. Their data and analyses demonstrate that chromatic aberration-corrected data collections should be performed at or near focus to enable constructive incorporation into subsequent reconstructions. The reduced contrast inherent in close-to-focus data collection is hoped to be recoverable with rapidly developing laser phase-plate technology (Wu *et al.*, 2025 ▸; Schwartz *et al.*, 2019 ▸).

The effect of inelastically scattered electrons is threefold. Firstly, inelastic scattering deposits energy in the sample, which leads to radiation damage. Secondly, the inelastically scattered electrons can increase the angular range of both the incident and elastically scattered beams, leading to a loss of signal, for example when the inelastically scattered electrons cannot be incorporated into a Bragg spot. Finally, inelastically scattered electrons can lead to an increase in the image background through ‘chromatic blur’. The deposition of energy and the attenuation of the signal occur irrespective of the use of energy filtering.

An alternative form of an information coefficient, based on the reciprocal of the damage-limited resolution, is discussed in Egerton & Nave (2026 ▸).

### 3.1. Contrast in electron microscopy

In electron microscopy, contrast arises from variations in intensity, which is proportional to the square of the amplitude of the wavefunction. This amplitude can originate from either phase contrast or amplitude contrast, each of which are governed by distinct interactions between the electron beam and the sample.

Phase contrast results from phase shifts in the electron waves as they transverse regions of varying electrostatic potential within the sample. These shifts lead to interference between differently phased wavefronts, producing contrast. This mechanism is essential in conventional transmission electron microscopy for imaging internal structures that lack significant differences in mass or thickness, such as biological specimens composed mainly of light elements (for example carbon, nitrogen and oxygen). Phase contrast is also the dominant mechanism underpinning electron diffraction from crystals and in ptychography, where interference patterns encode structural information. Phase contrast originates predominantly from elastic scattering, which preserves both the energy and coherence of the electron wave. Inelastic scattering disrupts coherence by removing electrons from interference pathways, such as Bragg reflections, thereby degrading phase information and reducing coherent contrast.

Amplitude contrast arises from spatial variations in how different regions of a sample scatter or absorb the incident electron beam. It includes several mechanisms: mass-thickness contrast, *Z* (atomic number)-contrast, scattering contrast, diffraction contrast and absorption contrast. Mass-thickness contrast occurs when thicker or denser regions scatter more electrons away from the optical axis, and these high-angle scattered electrons are often removed using a small objective aperture in the back focal plane of the microscope objective lens, which results in lower intensities in those areas. *Z*-contrast similarly arises from increased scattering by atoms with higher atomic number. The resulting intensity differences are governed by variations in the scattering cross section, which depend on atomic number, density and thickness. Scattering contrast more broadly refers to differences in image intensity caused by variations in both elastic and inelastic scattering across the sample. In high-angle annular dark-field scanning transmission electron microscopy (HAADF STEM), for example, scattering contrast dominated by incoherent elastic scattering at large angles produces *Z*-contrast images with intensity approximately proportional to the square of the atomic number. Diffraction contrast, another subtype of amplitude contrast, arises from coherent elastic scattering and is particularly relevant in crystalline materials. When the Bragg condition is satisfied, electrons are diffracted by specific lattice planes, producing intensity variations due to constructive or destructive interference. The strength of these diffracted beams depends on crystal orientation, strain and defects, and this mechanism is central to techniques such as selected-area electron diffraction (SAED) and convergent-beam electron diffraction (CBED). If the electrons undergo elastic or inelastic scattering to undetectable angles then this can be treated as absorption contrast, for example in contributing to the term *T* in the information coefficient in Peet *et al.* (2019 ▸). Absorption contrast, on the other hand, results from attenuation of the electron beam as it passes through the sample. Thicker or denser regions absorb more electrons, reducing transmitted intensity according to the Beer–Lambert law. In electron microscopy, absorption contrast is generally much weaker than scattering contrast and is often negligible in thin specimens.

In conventional STEM, the sample is positioned near the beam focus and contrast is recorded using one of the following.

(i) A bright-field (BF) detector, which collects unscattered or weakly scattered electrons.
(ii) An annular dark-field (ADF) detector, which collects both elastic and inelastically scattered electrons at intermediate angles. It is possible to record only the inelastically scattered electrons using energy filtering (EFTEM) although, due to delocalization at small energy losses (Muller & Silcox, 1995 ▸; Egerton, 2018 ▸), the data would not extend to as high a resolution as amplitude contrast from elastic scattering.
(iii) HAADF detectors, which collect strongly scattered or incoherently scattered electrons at high angles, emphasizing *Z*-contrast.

Recent advances in detector technology have enabled 4D STEM, where a pixelated detector records a full diffraction pattern at each scan point. This produces a four-dimensional dataset (2D probe position × 2D diffraction pattern), allowing flexible post-processing to extract the BF and (HA)ADF regions, and even phase-contrast signals. In principle, 4D STEM approaches enable contrast to be optimized across varying specimen thicknesses.

A recent paper (Rez *et al.*, 2025 ▸) gives a comprehensive description of the factors which maximize the contrast in imaging thick biological samples by electron microscopy and discusses different regimes for imaging with 200 keV electrons using amplitude and/or phase contrast as discussed above. These regions are the following.

(i) A thin weak phase object, such as in single-particle imaging of proteins, with a sample thickness limit of approximately 50 nm.
(ii) A strong phase object limited by attenuation from inelastic scattering for both TEM, with a sample-thickness limit of 200 nm.
(iii) Similar to (ii) but a mixed amplitude/phase object imaged using STEM with a sample-thickness limit of approximately 600 nm due to attenuation from large-angle elastic scattering.
(iv) Totally incoherent imaging using STEM with the bright-field collection aperture set to maximize the signal-to-noise ratio (Rez *et al.*, 2016 ▸).

### 3.2. Mean free paths for elastic and inelastic scattering

The concept of mean free path (MFP), defined as the average distance a moving particle travels before undergoing a change in direction or energy, is fundamental to understanding how sample thickness and composition influence contrast or the achievable information coefficient at a given incident electron energy. For a given sample, the elastic and inelastic MFPs, denoted as λ_e_ and λ_i_, respectively, can be calculated as
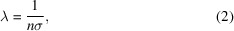
where σ is the scattering cross section (nm^2^) and *n* is the number density in atoms per volume (*n* = ρ*N*_A_/*M*), where ρ is the bulk density (g nm^−3^), *N*_A_ is Avogadro’s number (mol^−1^) and *M* is the molar mass (g mol^−1^). The elastic and inelastic cross sections in nm^2^, σ_e_ and σ_i_, respectively, can be estimated using expressions in Langmore & Smith (1992 ▸):
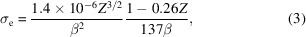

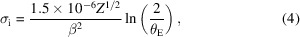

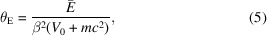
where *Z* is the atomic number and β is the velocity of the electron divided by that of light {β^2^ = 1 − [*mc*^2^/(*V*_0_ + *mc*^2^)^2^]}, θ_E_ is the characteristic angle for inelastic scattering, 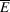 is the average energy loss, *V*_0_ is the electron acceleration voltage and *mc*^2^ is the rest energy of the electron. The characteristic angle defines the angular width of the scattered electron distribution for a specific energy loss. The MFP values reported in Table 1 ▸ are based on a prototypical protein crystal with 50%(*v*/*v*) solvent content, defined by the fractional atomic composition H_0.57_C_0.17_N_0.05_O_0.20_S_0.01_ (Latychevskaia & Abrahams, 2019 ▸). The elastic and inelastic MFPs for this composition were calculated using the framework of Langmore & Smith (1992 ▸). It should be noted that IMFP (inelastic mean free path) values obtained using this approach are often slightly lower than those estimated from *ESTAR* (Berger *et al.*, 2005 ▸). *ESTAR*-based calculations typically yield longer MFPs than Langmore–Smith for the same material and energy, primarily due to differences in how inelastic scattering is modelled. This variability is visualized in Supplementary Fig. S1, which compares IMFPs for carbon, vitreous ice and a prototypical protein across ESTAR and Langmore–Smith models. The effect of solvent content on the IMFP is illustrated in Supplementary Fig. S2, which shows IMFPs computed for protein crystals with 30–80% water content using Langmore–Smith. The variation in IMFP with solvent content is modest compared with method-dependent differences.

### 3.3. Coherence effects in electron diffraction

Electron diffraction, as a phase-contrast technique, relies on elastically scattered electrons. If both elastic and inelastic scattering data are recorded, inelastically scattered electrons can be treated as a loss of both temporal coherence (change in energy Δ*E*) and spatial coherence (change in angle θ_E_) of the incident electron beam, as discussed in Rez (2023 ▸).

The inelastic scatter of electrons by plasmons has an energy loss of 20–25 eV (Egerton, 2017 ▸). This energy loss is relatively small, meaning that for a 200 keV electron beam the change in longitudinal coherence can be neglected. However, the effects of a change in transverse coherence can be much more significant, as shown in Table 2 ▸.

For a typical crystal of a small molecule, the characteristic angle is much smaller than the angular spacing between lattice points (see also diagram 2 of Rez, 2023 ▸) and it might be possible to include the inelastic scattering in the estimation of the spot intensity, for example by careful setup of an energy filter. However, a lattice spacing of 10 nm, typical of a protein crystal, corresponds to an angle of 0.25 mrad (at 200 keV), with each of the diffraction spots having a ‘halo’ round them of half angle 0.073 mrad (total spread 0.146 mrad) due to inelastically scattered electrons. The effect of the inelastic scattering therefore extends most of the way between the spots. There is an advantage in going to higher energy as the half angle for inelastic scattering covers 30% of the angular distance between 10 nm spots at 200 keV and only 22% at 1 MeV. The volume of the halo in the three-dimensional diffraction pattern is a factor of 2.5 smaller at 1 MeV, perhaps allowing coarser energy filtering to be used.

Protein crystals usually have a large amount of disordered solvent and exhibit multiple conformations of solvent-exposed side chains, which contribute to background. This heightened background can be mitigated by setting up the instrument to give sharp diffraction spots, limited by crystal perfection. This includes optimizing parameters such as incident beam divergence, spectral bandwidth, detector distance and beam size at the specimen, as described for X-rays in Nave (2014 ▸). An increase in the breadth of the diffraction spots is therefore likely to result in poorer resolution data from protein crystals. Multiple inelastic scattering will result in further broadening. In addition, there are large Lorentzian tails on the angular distribution of inelastically scattered electrons (Egerton, 1976 ▸), as described in Section 3.5. As the inelastic scattering does not contribute usefully to the Bragg spots, the Peet *et al.* (2019 ▸) information coefficient in equation (1) will hold for the case of electron diffraction from protein crystals, with a modification for the number of unit cells *N* along the beam path. The proposed information coefficient is
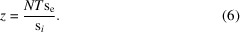
This modification, which applies to needle-shaped crystals, does not affect the relative information coefficient as a function of electron energy for a defined thickness of crystal. However, it does boost the information coefficient. For crystals that are the same size in each dimension a factor of up to *N*^3^ could be applied.

A recent paper by Dickerson *et al.* (2024 ▸) analyses the information coefficient for electron crystallography and concludes

the peak information coefficient increases as the energy increases, with the overall highest being with an 820 keV beam and a 250 nm thick crystal. At energies above this, the peak information coefficient begins to reduce, and there is little or no extra improvement for crystals <1 µm thick.

The energy value for the peak information coefficient is similar to the value of 700 keV calculated by Rez *et al.* (2025 ▸) and is a manifestation of the MFPs saturating at these energies, as shown in Fig. 2 of Rez *et al.* (2025 ▸). The metric discussed in Dickerson *et al.* (2024 ▸) is also similar to equation (6) and would also scale by *N*^3^ in the 3D case when the exposed area is increased to cover the crystal (Joshua Dickerson, personal communication).

For perfect crystals the diffraction spots become sharper as the number of unit cells in the beam increases. This has been described as a Bragg boost (Spence, 2017 ▸; Egerton & Nave, 2026 ▸) and means that the diffraction spots are more easily detected above the background from disordered material in the crystal, potentially leading to higher resolution data. This effect might shift the 250 nm optimum for electron diffraction to larger crystals. This would depend on the extent of the intrinsic background from the crystal and the extent to which the data-collection procedures are matched to the crystal perfection.

The background contribution due to inelastic scattering becomes progressively worse as the unit-cell dimensions increase. This is because for larger unit cells at the same resolution and detector size, the distance between spots will cover progressively fewer pixels and the contribution of the background to each spot will therefore increase. Sources of background include, but are not limited to, the following.

(i) Background from the instrument, including detector noise. The latter is becoming less of an issue with the advent of modern direct electron-counting detectors.
(ii) Background from water/mother liquor surrounding the crystal.
(iii) Background from the crystal due for example to disordered water and side chains. This background can increase due to radiation damage.
(iv) Background from interaction of the incident radiation on the crystal (for example due to inelastic scattering and multiple elastic scattering).

The first two categories of background could be mitigated by improvements in instrumentation and sample preparation, while energy filtering could specifically reduce the background caused by inelastic scatter. As for X-ray crystallography, matching the instrumental setup to the properties of the protein crystal, including the crystal imperfections, should maximize the signal. A general review of nano crystallography, emphasizing the effect of crystal imperfections, is given in Vlahakis *et al.* (2024 ▸).

### 3.4. Multiple scattering of electrons

The small values of electron MFPs mean that multiple scattering is likely in the specimen size ranges considered. Inelastic scattering will reduce the coherence length of the X-rays, resulting in both a broadening of the spots on a detector and an increase in the angular reflecting range of the reflections. The effect is to increase the spot size in all three directions of the reciprocal-space diffraction pattern. This will only become apparent if the data are recorded with sufficiently fine rotation increments using a detector with sufficient pixels. Scattering angles and energy losses for inelastically scattered electrons are given in Rez (2023 ▸) and a discussion of the contribution of inelastically scattered electrons to image formation is included in Dickerson & Russo (2022 ▸). These considerations apply to both electron crystallography and electron imaging using ptychography.

As well as inelastic scattering, the effects of multiple elastic scattering, which can be modelled by multi-slice methods (Cowley & Moodie, 1957 ▸), must be considered. For crystals, there is the additional possibility of strong multi-beam effects. The term dynamic diffraction is reserved for this effect in crystals to distinguish it from general multiple scattering. Dynamic diffraction can change the observed diffraction intensities and lead to a breakdown in Friedel’s law. In addition, intensity can be observed for otherwise forbidden reflections, for example when a multiple diffracted beam occurs in the direction corresponding to systematic absences which depend on the crystal orientation. The possibility of dynamic diffraction also depends on the MFP for elastic scattering, the crystal volume, the cell volume, the magnitude of the structure factors, the space group and the crystal perfection, including the mosaicity.

Seminal work on crystal lamella demonstrated that interpretable data could be obtained from electron diffraction (Duyvesteyn *et al.*, 2018 ▸; Parkhurst *et al.*, 2023 ▸; Clabbers & Gonen, 2025 ▸). For lamella, consideration should be given to the increase in path length as the sample is tilted in the beam. For more isometric crystals, Shi *et al.* (2013 ▸) found that interpretable electron diffraction data could be obtained from lysozyme crystals 0.5 µm in size using an electron energy of 200 keV. Weak forbidden reflections were observed and interpreted as due to dynamic interactions (multiple beam diffraction).

A general discussion of dynamic diffraction, applying to X-rays, neutrons and electrons, is given in Sabine (2006 ▸). An article by Subramanian *et al.* (2015 ▸) used simulations to suggest a thickness limit of 100 nm for electron diffraction of perfect protein crystals (defined as an *R* factor < 0.3). They hypothesize that it is crystal mosaicity and bending that allow protein structures to be solved from thicker crystals. However, they also point out that nanocrystals with a large unit cell produce weaker diffracted beams for the same crystal volume. This would mean that, other things being equal, the effects of dynamic diffraction would be weaker for crystals with larger unit cells than the lysozyme example discussed by Shi *et al.* (2013 ▸). Multiple scattering will still occur for the same size crystal with larger unit cells according to the elastic MFP, but will result in much smaller errors distributed among many reflections. Dynamic diffraction can be minimized by careful orientation of the electron beam to the crystal during data collection, and precession methods are increasingly being used to achieve this (Mugnaioli *et al.*, 2009 ▸). The MFP for elastic scattering of electrons in a prototypical protein crystal with 50%(*v*/*v*) solvent content (defined as the fractional atomic composition H_0.57_C_0.17_N_0.05_O_0.20_S_0.01_ by Latychevskaia & Abrahams, 2019 ▸) is 324 nm at 200 keV, rising to 403 nm at 1 MeV, using equation (3) (see Table 1 ▸).

Holton & Frankel (2010 ▸) developed a theoretical model for the X-ray scattering power of protein crystals based on the atomic composition, the crystal volume, the unit-cell volume and the associated scattering power.

For evaluating the dependence on the unit-cell volume, a simplified version of equation (1) from Holton and Frankel gives the intensity of a reflection as
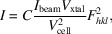
where *I*_beam_ is the intensity of the incident beam, *V*_xtal_ is the volume of the crystal, *V*_cell_ is the volume of the unit cell, 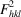
is the square of the structure factor and *C* includes constant factors such as the wavelength and Lorentz and polarization corrections. Holton and Frankel conclude that at resolutions common in crystallography, the square of the average structure factor is proportional to *V*_cell_ when the number of atoms per unit volume is fixed. For the same sized crystal, this cancels out one of the *V*_cell_ terms, meaning that the value of *I* is proportional to *V*_xtal_/*V*_cell_. This leads to a strong dependence of the integrated intensity on the cell dimensions and means that, other things being equal, a protein crystal with larger unit-cell dimensions than the lysozyme example of Shi *et al.* (2013 ▸) will give much less dynamic scattering.

The above applies to 3D crystals. Dynamic effects, including a breakdown of Friedel’s law, have also been observed for 2D crystals of bacteriorhodopsin (Glaeser & Ceska, 1989 ▸). The diffraction pattern of such crystals is a continuous intensity normal to the plane of the crystals but crystal-like diffraction when projected onto the plane. This effectively means that the conditions for dynamic diffraction will occur independent of any crystal orientation, although it would not be expected to be as strong as a 3D crystal in orientations which maximize the dynamic scattering. Similar considerations which apply to 2D crystals should also apply to fibre samples and imperfect crystals, where the diffraction spots are broadened, leading to an increased probability of weak dynamic diffraction.

It is possible in principle to calculate the effects of dynamic scattering and correct for them. An extreme example (for crystals of silicon) is given in Cleverley & Beanland (2023 ▸). Such an approach might also be possible for small-molecule crystallography. To observe dynamic scattering and compensate for the effects, it is important to collect the data in fine rotation increments. This method has the additional benefit of providing a better signal to background and should always be performed if a detector with a low readout noise is available.

### 3.5. Implications for electron ptychography, STEM and 4D STEM

The principles and advantages of electron ptychography are well established (Humphry *et al.*, 2012 ▸) and the current highest resolution data acquired with electrons rely on the method (see, for example, Li *et al.*, 2025 ▸). Ptychographic measurements record the continuous scattering pattern from elastically scattered electrons. For thin specimens (or thicker if using higher energy electrons) inelastic scattering is minimal, and ptychography with or without an energy filter and a suitable distance to the focal plane is appropriate. The effect of inelastic scattering is to create a halo of half width θ_E_ centred around a diffraction peak. In addition to this halo, there will be a long Lorentzian tail. The median scattering angle is around 10θ_E_ and the mean scattering angle is around 22θ_E_ (Egerton, 1976 ▸). These inelastically scattered electrons will add to the background. For imaging, there might be an advantage in collecting data for which the halo is contained within the first Fresnel zone of the elastic scattering, in a similar approach to utilizing the halo round a diffraction spot from a crystal. The angle of the first Fresnel fringe (for a parallel beam) is given approximately by 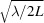, where *L* is the distance to the detector. For cone beam illumination, the Fresnel scaling theorem could be used to convert the arrangement to that for a parallel beam. The distance of the sample from the focus also influences the magnification, which is a critical parameter for matching the diffraction image scale to the detector pixel size.

For specimens of significant thickness, a conventional STEM approach can be used, with the specimen placed near the focus and utilizing bright-field and annular dark-field detectors to exploit both elastic and inelastic scattering. The size of the beam at the specimen is at a minimum at a slight negative defocus (see Fig. 3 in Egerton & Watanabe, 2022 ▸). Retaining the use of two-dimensional (2D) detectors would still capture any coherent scattering information and provide greater flexibility in the virtual detector geometry for bright-field and dark-field imaging. As sample thickness increases it should be noted that the impact of beam broadening as the electron beam transits a thick specimen can be resolution-limiting (Egerton, 2017 ▸).

## 4. X-ray diffraction and imaging

For X-rays the main interactions are elastic scattering (giving phase contrast), Compton scattering and absorption via the photoelectric effect. Both absorption and Compton scattering lead to deposition of energy in the sample and consequent radiation damage. The contribution of Compton scattering to radiation damage in X-ray diffraction from crystals is included in the analysis by Cowan & Nave (2008 ▸) and is incorporated into the program *RADDOSE*-3*D* (Bury *et al.*, 2018 ▸). The contribution only becomes significant above 25 keV.

For X-ray imaging of cells and tissues, both absorption contrast and phase contrast in the water window (280–530 eV) are favourable. This is because the cross sections for both absorption and phase contrast by carbon are much higher than for the oxygen in the surrounding water. However, strong absorption limits the specimen thickness to about 10 µm. For thicker specimens, energies above 2 keV using phase contrast are more favourable. A comparison of phase and absorption contrast is given in Nave (2018 ▸) and Nave (2020 ▸), the latter also including an analysis of Compton scattering for imaging.

X-ray diffraction from crystals utilizes phase contrast. The diffraction spots are enlarged due to effects such as crystal size, crystal imperfections, incident beam divergence and detector pixel sizes. Ideally, all of these parameters should be matched (for a rather complex description of a way of achieving this, see Nave, 2014 ▸). For the present purpose, it is assumed that the diffraction spots remain sharp, leading to an optimum signal-to-background ratio, and that the X-ray energy is chosen to give a high transmission (*T* ≃ 1), an easy thing to do for crystals of a few micrometres in thickness. In addition, higher energy X-rays should produce less radiation damage in crystals of a few micrometres in size due to photoelectron escape (Nave & Hill, 2005 ▸; Cowan & Nave, 2008 ▸). This requires care in crystal mounting and, in practice, improvements by factors of 2–3 have been observed (Storm *et al.*, 2020 ▸). The benefits of matching the beam size at the crystal to that of the crystal are demonstrated in Warren *et al.* (2024 ▸).

## 5. X-rays and electrons compared

Comparisons between electrons and X-rays for imaging frozen-hydrated organic materials in the presence of radiation damage are given in Rez (2021 ▸), Du & Jacobsen (2018 ▸) and Egerton & Nave (2026 ▸).

For thin samples, Henderson (1995 ▸) calculated that electrons have a factor of at least 500 advantage over X-rays in terms of dose deposited per elastic scattering event. For thicker specimens this must be multiplied by the transmission factor, which can be assumed to be 1 for X-rays. The crossover point between electrons and X-rays from the point of view of radiation damage must take account of the factor of 500 advantage for electron scattering for the same dose together with the increased transmission for X-ray scattering. This means that the crossover point will be at a value of *T* of 1/500 for electron scattering or perhaps 1/300 if photoelectron escape can be exploited for X-rays. The crossover points (Table 3 ▸) were calculated from the crystal thickness when exp(*p*) equals 500 (giving a transmission *T* of 0.02), where *p* is the number of total MFPs within the path of the beam in the crystal, assuming a prototypical protein crystal with 50%(*v*/*v*) solvent content.

Scaling from experimental data (right column in Table 3), one might expect interpretable data for crystals over 700 nm in thickness at 1000 keV, a 43% increase in thickness compared with 300 keV. Developments in electron crystallography using submicrometre crystals are continuing (Fiorini *et al.*, 2026 ▸). Yonekura *et al.* (2002 ▸) demonstrated a factor of 5.5 improvement in the background at 0.3 nm resolution using a 10 eV energy filter for 60–75 nm thick crystals, and significantly increased benefits should be obtained for even thicker crystals. However, better data for electron crystallography alone would still be obtained from 250 nm crystals (Dickerson *et al.*, 2024 ▸; see Section 3.3).

Collection of X-ray diffraction data from multiple micrometre-sized crystals distributed on an electron microscopy grid is becoming routine and this is transferring to electron diffraction (Hofer *et al.*, 2025 ▸) for submicrometre crystals. In some cases (for example the study of crystal phases in pharmaceutical tablets) the choice of crystal size is fixed by the sample and the study of micrometre-sized crystals by electron diffraction would have a role.

## 6. Identifying regions of interest: proteins and cellular organelles

Multimodal or correlated imaging combines data from more than one imaging technique applied to the same sample. A typical example is to combine fluorescent imaging to locate specific features such as a labelled virus or protein with X-ray and/or electron imaging to obtain details about the whole sample. This includes the possibility of surveying large, native specimens using a fast technique such as imaging with X-rays to identify regions of interest. These regions can then be studied by higher resolution slower techniques such as electron imaging of thin sections extracted from the sample. An example would be to identify the regions of the cell or tissue where there is a high concentration of infecting virus particles. These regions could then be extracted for higher resolution investigations by electron microscopy to obtain details of the binding of the virus to specific cellular components. There will however be a maximum dose budget that will have to be shared between the two imaging methods. It is likely that many structural biologists will be reluctant to use more than 10–20% of the tolerable dose for identifying a region of interest. The discussion below does not include this factor, which will have to be applied for each case. The radiation dose limit for electrons and X-rays is similar (see, for example, Hitchcock *et al.*, 2008 ▸).

There are two possible requirements for high-resolution electron tomography. Firstly, one might want to identify the presence and orientation of a particular protein bound to a cellular organelle or another protein. This may require preservation of the structure at a resolution where secondary-structural elements could be observed (approximately 1.0 nm for distances between α-helices and 0.5 nm for distances between β-sheets). In addition, there is a requirement to refine structures within cells using subtomogram averaging. This ideally requires preservation of the structure at a resolution corresponding to van der Waals distances (0.3–0.5 nm) or, ideally, covalent bond distances (0.1–0.2 nm).

**Figure 1:**
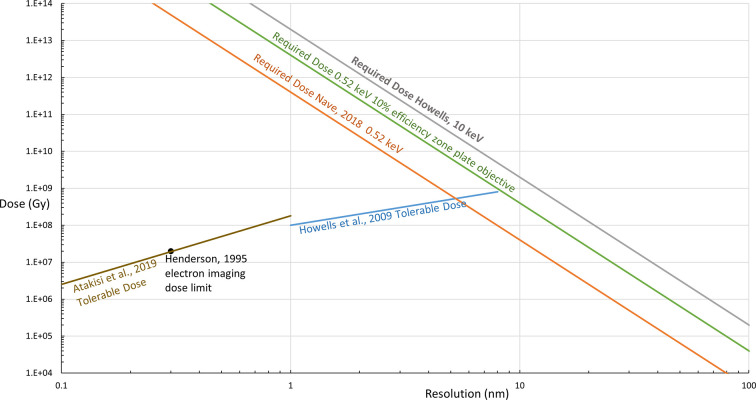
Values for required dose (lines sloping upwards from right to left) against resolution using phase contrast to identify a protein within the water window at 0.52 keV (Nave, 2018 ▸) and at 10 keV (Howells *et al.*, 2009 ▸) with 100% data-collection efficiency. The required dose assuming a 10% efficiency zone-plate objective operating at 0.52 keV is also shown. Plots for the resolution dependence for the tolerable dose (lines sloping upwards from left to right) at which the intensity decreases by half are shown with a 100 MGy nm^−1^ dependence (Howells *et al.*, 2009 ▸) and a *q*^1.86^ dependence (Atakisi *et al.*, 2019 ▸), where *q* is the wavevector (2π/*d*).

Fig. 1 ▸ illustrates the required dose in X-ray imaging to detect the presence of proteins of various sizes, and the dose-limited resolution achievable when determining the structure of proteins with electrons.

As an example, if one wants to identify the presence of a 10 n*M* protein using X-rays and phase contrast within the water window, the required dose would be 4 × 10^7^ Gy (see the line in Fig. 1 ▸ labelled Nave, 2018 0.52 keV) assuming 100% data-collection efficiency and no loss of signal due to absorption. This dose figure is similar to the maximum tolerable dose for protein structure determination at 0.5 nm resolution. Estimates of the required dose using 10 keV X-rays for a 10 nm diameter protein vary between 2 × 10^9^ Gy (Fig. 2*b* of Howells *et al.*, 2009 ▸) and 4 × 10^8^ Gy (Fig. 11 of Du & Jacobsen, 2018 ▸). The differences are likely to be due to the use of alternative criteria for defining the densities of the protein and surroundings as well as varying criteria for defining the resolution. In any case, these estimates are beyond the tolerable dose for electron microscopy at 0.5 or 0.25 nm resolution. For a 30 nm diameter protein the required dose would decrease to approximately 5 × 10^5^ Gy at 0.52 keV, much less than the tolerable dose for electron imaging at 0.5 or 0.25 nm. The required dose for a 30 nm virus particle is likely to be similar.

Fig. 1 ▸ is a simplified version of one given in Nave (2020 ▸) which includes the required dose for identifying various cellular organelles using both phase and absorption contrast. A list of cellular organelles is given in that paper, illustrating a wide variation for the required dose.

For thin specimens, both phase and absorption contrast at higher energies require much higher doses than phase or absorption contrast in the water window to obtain the same signal. If using a zone-plate objective, the overall efficiency of the zone plate must also be considered. Zone-plate efficiencies are discussed in Wu *et al.* (2012 ▸) and Li & Jacobsen (2018 ▸). Higher resolution zone plates require a higher aspect ratio to achieve high efficiencies and are more demanding to fabricate. Typical objective zone-plate efficiencies are around 10%, meaning that their use requires ten times more fluence and dose, as shown by the green line in Fig. 1 ▸. These considerations do not apply to imaging using ptychography or similar coherent imaging methods, although other losses could occur in these cases. For thicker specimens (*e.g.* 10 nm or greater) the attenuation through the sample when operating in the water window becomes severe and phase-contrast techniques using higher energy X-rays become more favourable, as discussed in Egerton & Nave (2026 ▸).

A recent paper (Blum *et al.*, 2026 ▸) investigated the possibility of using X-ray imaging to identify regions of interest for subsequent examination by electron microscopy. An electron microscopy grid containing apoferritin molecules was exposed to 13.5 keV X-rays at doses between 1 and 100 MGy. Subsequent electron microscopy showed that resolutions of 3.1–3.7 Å could be obtained compared with a resolution of 3.1 Å for a portion of the grid not exposed to X-rays. It should be noted that the final resolution achieved for the cryoEM ‘industry-standard’ sample apoferritin was relatively low and that the grid was severely damaged during transport between facilities so the impact of the absorbed X-ray dose may have been significantly greater than is reported in this analysis. A related analysis by Groen *et al.* (2025 ▸) compared radiation damage in electron imaging of biological tissues with or without previous exposure during full-field imaging with soft X-rays. These preliminary results were consistent with the analysis above.

The discussion for X-ray imaging above relates to the required dose to identify specific features and the consequence for higher resolution electron microscopy. The consequence of radiation damage on the X-ray images has been discussed by Du & Jacobsen (2018 ▸). This includes ‘However, microscopy at tens of nanometres spatial resolution is limited not by bond breaking but by mass loss or rearrangement at much longer length scales…’. It is assumed that mass loss will severely affect the possibility of subsequently examining the sample by electron microscopy. However, rearrangement on much longer length scales need not result in disorder at spatial frequencies corresponding to the dimensions of cellular organelles. It has been possible to correct for long-range rearrangements over tens of micrometres in stained and fixed brain tissue and still retain the information about synapses at length scales of 35 nm (Bosch *et al.*, 2025 ▸). In a similar manner, long-range rearrangements due to radiation damage can also affect unit-cell spacings in protein crystals and such effects are not well correlated with loss of resolution (Ravelli *et al.*, 2002 ▸). The developments in expansion microscopy (summarized in Wassie *et al.*, 2019 ▸) show that long-range rearrangements can still preserve local structure.

## 7. Conclusion

Another paper (Egerton & Nave, 2026 ▸) in this special issue discusses the damage-limited resolution for both X-ray and electron imaging. Where there is overlap, the results are broadly consistent, given the differences in various parameters such as the atomic composition and density of various features and the precise means of defining resolution.

The information coefficient is a useful metric for comparing the performance of different electron energies for both thin and thick specimens. It is applicable to single-particle imaging, electron diffraction and electron ptychography. The comparison with X-rays indicates that the crossover point between X-rays and electrons could occur for quite thick specimens. This indicates that electrons are superior to X-rays for such thick specimens based on the information coefficient alone. Better data could be obtained from smaller crystals by electron diffraction or by X-ray diffraction from multiple micrometre-sized crystals. For the same size crystal, collecting data by electron diffraction for smaller sized unit cells is less likely to result in significant multibeam diffraction effects compared with that for larger unit cells. There is also a benefit in operating at higher energies of up to nearly 1 MeV. These benefits will be fully explored and validated with the new High Energy Xtallography Instrument (HeXI), a dedicated high-energy electron diffractometer with a tunable electron source operating between 100 kV and 1 MeV, currently under construction at Diamond Light Source. HeXI leverages MX-grade goniometry together with the increased penetrating power of MeV electrons to enable high-fidelity structure determination from crystals in the 300 nm to 3 µm size range and is scheduled to open for user commissioning in 2027. For frozen-hydrated specimens, the possibility of finding regions of interest for subsequent examination at higher resolution by electron microscopy indicates that this should be possible at an X-ray resolution of around 30 nm, the size of a small virus particle. However, there are still some developments required to achieve this.

One of the most important factors limiting data quality for both imaging and diffraction, with X-rays or electrons, is sample preparation and handling. Optimizing this, together with improvements in instruments and analysis, should enable a closer convergence with some of the ideal assumptions assumed in the present paper.

## Supplementary Material

Supplementary Figures. DOI: 10.1107/S2059798326002056/gm5119sup1.pdf

## Figures and Tables

**Table 1:** The values of the elastic and inelastic MFPs (in nm) for a protein crystal with a fractional atomic composition H_0.57_C_0.17_N_0.05_O_0.20_S_0.01_

Energy (keV)	Inelastic MFP (nm)	Elastic MFP (nm)
100	99	202
200	150	324
300	182	403
1000	244	590

**Table 2:** Comparison between the characteristic angle for inelastic scattering and typical angular spacings between lattice points for a small-molecule crystal and a protein crystal

Energy (kV)	Inelastic scattering characteristic angle (Δ*E* = 25 eV) (mrad)	Small-molecule (1 nm unit cell) lattice spacing (mrad)	Protein (10 nm unit cell) lattice spacing (mrad)
100	0.136	3.70	0.37
200	0.073	2.51	0.25
300	0.051	1.97	0.19
1000	0.018	0.87	0.08

**Table 3:** Total MFPs for a prototypical protein crystal with 50%(*v*/*v*) solvent content, calculated using equations (3) and (4), and crossover points for crystal thickness where the information coefficient for electron and X-ray crystallography is the same X-ray crossover points would be approximately 10% smaller if photoelectron escape is included.

Electron energy (keV)	Total mean free path† (nm)	X-ray crossover‡ (µm)	Experimentally scaled crossover§ (nm)
200	474.1	2.95	446.3
300	584.4	3.63	550.0
1000	833.6	5.18	784.6

† Prototypical crystal with 50%(*v*/*v*) solvent content.

‡ Neglects absorption and multiple scattering effects not included.

§ ED data-processing limit of 550 nm at 300 keV from Martynowycz *et al.* (2021 ▸), without the use of energy filters.
