# Supplementary material for: Electrons and X-rays for diffraction and imaging
Source: Acta Crystallogr D Struct Biol. 2026 Apr 7;82(Pt 5):446–56. doi: 10.1107/S2059798326002056 (PMC13133989; doi:10.1107/S2059798326002056)
Supplement: Supplementary file 1 [file d-82-00446-sup1.pdf]

STRUCTURAL
BIOLOGY

Volume 82 (2026)

Supporting information for article:

Electrons and X-rays for diffraction and imaging

Colin Nave, Pedro Nunes and Alistair Siebert

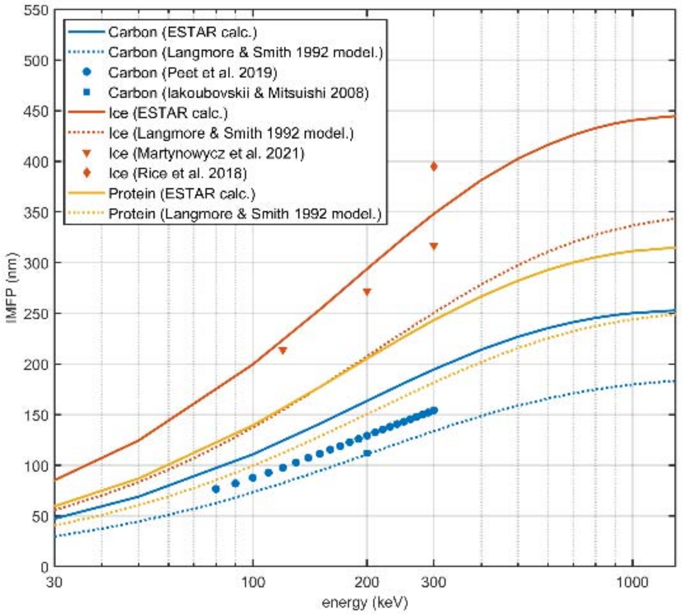

Figure S1 Comparison of IMFP for carbon, ice and a prototypical protein across ESTAR (Berger et al., 2005) and Langmore and Smith (1992) models with selected literature values.

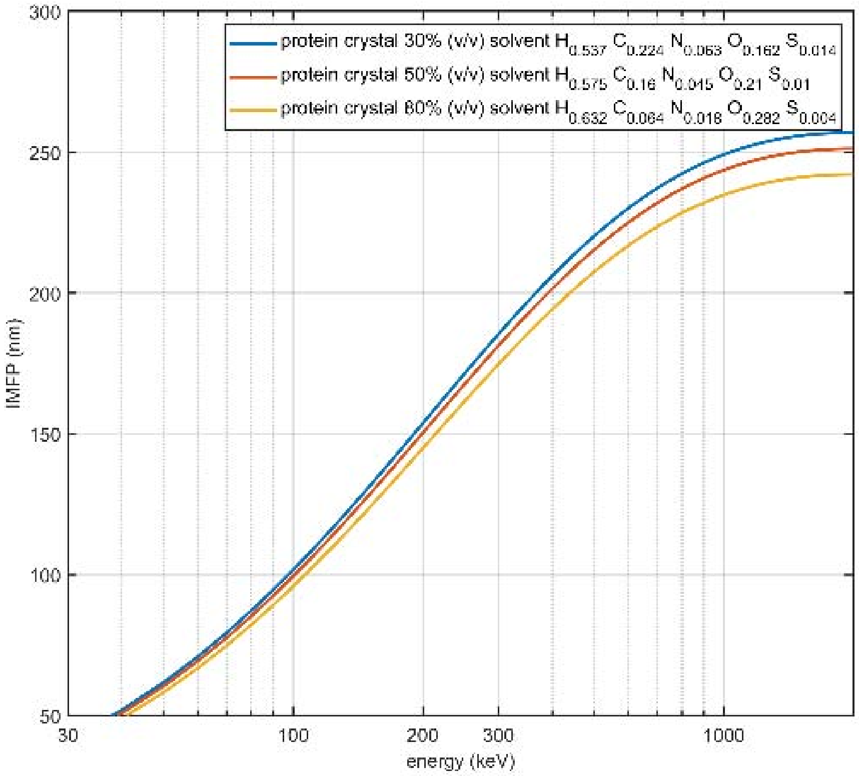

Figure S2 Comparison of IMFP (inelastic mean free path) for a prototypical protein assuming solvent contents spanning 30–80%.
